# Dogs with separation-related problems show a “less pessimistic” cognitive bias during treatment with fluoxetine (Reconcile™) and a behaviour modification plan

**DOI:** 10.1186/s12917-015-0373-1

**Published:** 2015-03-28

**Authors:** Christos I Karagiannis, Oliver HP Burman, Daniel S Mills

**Affiliations:** Animal Behaviour, Cognition and Welfare Group, School of Life Sciences, University of Lincoln, Lincoln, LN6 7DL UK

## Abstract

**Background:**

Canine separation-related problems (SRP) (also described as “separation anxiety” or “separation distress”) are among the most common behavioural complaints of dog owners. Treatment with psychoactive medication in parallel with a behaviour modification plan is well documented in the literature, but it is unknown if this is associated with an improvement in underlying affective state (emotion and mood) or simply an inhibition of the behaviour. Cognitive judgement bias tasks have been proposed as a method for assessing underlying affective state and so we used this approach to identify if any change in clinical signs during treatment was associated with a consistent change in cognitive bias (affective state).

Five dogs showing signs of SRP (vocalising – e.g. barking, howling-, destruction of property, and toileting – urination or defecation- when alone) were treated with fluoxetine chewable tablets (Reconcile™) and set on a standard behaviour modification plan for two months. Questionnaires and interviews of the owners were used to monitor the clinical progress of the dogs. Subjects were also evaluated using a spatial cognitive bias test to infer changes in underlying affect prior to, and during, treatment. Concurrently, seven other dogs without signs of SRP were tested in the same way to act as controls. Furthermore, possible correlations between cognitive bias and clinical measures were also assessed for dogs with SRP.

**Results:**

Prior to treatment, the dogs with SRP responded to ambiguous positions in the cognitive bias test negatively (i.e. with slower running speeds) compared to control dogs (p < 0.05). On weeks 2 and 6 of treatment, SRP dogs displayed similar responses in the cognitive bias test to control dogs, consistent with the possible normalization of affect during treatment, with this effect more pronounced at week 6 (p > 0.05). Questionnaire based clinical measures were significantly correlated among themselves and with performance in the cognitive bias test.

**Conclusion:**

These results demonstrate for the first time that the clinical treatment of a negative affective state and associated behaviours in a non-human species can produce a shift in cognitive bias. These findings demonstrate how the outcome of an intervention on a clinical problem can be evaluated to determine not only that the subject’s behaviour has improved, but also its psychological state (welfare)

**Electronic supplementary material:**

The online version of this article (doi:10.1186/s12917-015-0373-1) contains supplementary material, which is available to authorized users.

## Background

Separation-related problems (SRP) in dogs are a common behavioural complaint of owners [1-3]. Approximately 14%-20% of dog patients [4,5] from general veterinary practice show SRP signs such as vocalising, destruction of property, and toileting when alone [6]. However, it is suspected that up to 50-56% of the whole dog population may display clinical symptoms of SRP at some point in their life [7,8], which out of the total population of approximately 70 million dogs in US and 70 million in Europe [9,10] represents about 35 million dogs with SRP in the Europe and 35 million dogs in US.

Even though this behavioural condition is common, the underlying motivation(s) of SRP and the causal aetiology of this phenomenon are still a matter of debate [11-13]. It has been suggested that an animal’s affective state (emotional predisposition or moods) can be indirectly assessed through the use of cognitive bias (CB) testing [14]. CB testing is based on the finding that decision making is affected by background affective state and, in the case of ambiguous stimuli; individuals experiencing negative affect tend to make more negative judgements than those in a more positive emotional state [14-17], for example, anxious people tend to interpret ambiguous situations more unfavourably. In spatial CB tests, animals are trained to discriminate between bowls placed in two different locations, one with food present (R+), and one without food (R-). Subsequently, bowls were placed in ambiguous, unrewarded locations (‘probes’) between the learned locations. A more “optimistic” bias is reflected in running relatively faster to the ambiguous probes, and a more “pessimistic” bias inferred by the converse [17]. Recently, it has been found that dogs in rescue kennels with SRP-like behaviour tested on a modified spatial cognitive bias test appear to have more negative underlying affective state than dogs not showing this behaviour [18]. However, it is not known whether these findings are transferable to the clinical condition of household pets.

These cases can often be managed effectively with a behaviour modification plan plus a pharmaceutical adjunct, such as a selective serotonin reuptake inhibitor, like fluoxetine, or a non-specific serotonin reuptake inhibitor, like clomipramine [19-22]. There is strong evidence for an increased rate of response to the behaviour modification plan when such medication is included [19-21]. However, because serotonergic medication can cause a general inhibition of behaviour, especially at higher doses [23], it is not known if the combined treatment with medication is associated with a concomitant improvement in well-being; i.e. whether the programme including medication improves the underlying affective state of the animal or simply inhibits the problem behaviour.

The aim of the present study was therefore to investigate whether clinical improvement of SRP-afflicted dogs, during treatment with a combination of fluoxetine and a standard behaviour modification plan, resulted in measurable changes in CB tests consistent with either an improvement in underlying affective state or general motor inhibition. In the former, we would hypothesise that running speeds should increase due to greater “optimism”, whereas in the latter they would be expected to decrease as a result of behavioural inhibition.

## Methods

### Ethics statement

This research was approved by the School of Life Sciences Ethics Committee at the University of Lincoln, UK. The owners of all the dogs have given written informed consent for the use of their dogs in that research.

Since previous studies (e.g. [19]) have demonstrated a greater improvement in the problem using a behaviour modification plan combined with fluoxetine compared to a placebo control, and because the aim of this study was primarily to investigate the correlates of improvement when using fluoxetine in conjunction with a behaviour modification plan, a placebo was considered unnecessary as well as undesirable from an ethical perspective.

### Subjects

Two groups of dogs were recruited for the study, one with SRP, hereby referred to as the clinical group, and one without SRP or any other diagnosed anxiety problem, designated the control group (Additional file 1: Table S1). All dogs were assessed and diagnosed by a veterinary clinician specialising in behavioural medicine. The purpose of the control group was to provide a time related control for the dogs with SRP and to control for the effects of repeated CB testing in dogs, since it has been suggested that judgement CB tests may be subject to learning effects that limit repeatability [24].

Recruitment of dogs with SRP was through advertisements in the local media in the Lincoln UK area and through veterinary clinics in both London and Lincoln. Inclusion and exclusion criteria for study selection were predefined (Table 1). The recruitment period occurred from July 2010 until February 2011. In total, 22 dog owners expressed interest in taking part in the study. Among these, 14 owners were not willing to comply with the study protocol requirements. One dog was excluded before the beginning of the study due to a clinical history of seizures, and another was excluded during treatment for medical reasons (co-incidental gastric dilatation and volvulus). One dog was also excluded due to its low food motivation, making it difficult to train in the CB test. Dogs excluded from the study were provided with a behaviour consultation and modification plan, without psychoactive medication (unless the owner was willing to cover the medication’s cost) in accordance with the agreed management of subjects described in our submission for ethical approval. Therefore, the clinical group was composed of five dogs of various breeds with SRP, 3 males (2 neutered and one intact) and 2 females (both neutered). The average age of the dogs was 4.2 years (range 2–6.5 years) and they had been owned by their current owners for 2.4 years on average (range 2 months - 6 years). Their average body weight was 22.6 kg (range 11–30.7 kg).

**Table 1:** **Inclusion and exclusion criteria for dogs taking part in the study**

**Inclusion criteria**	**Exclusion criteria**
1. Been in owner’s possession for at least 2 weeks	1. Changes in household planned within the next 2 months, e.g. moving house, major change in household schedule
2. At least 6 months of age.	2. Clinical history of seizures
3. Body weight within range 4 kg to 48 kg inclusive.	3. Neutered within 1 month of the study
4. Subjected to at least four episodes of owner absence per week	4. Pregnant or lactating
5. Clinical signs of separation anxiety for at least 2 weeks	5. Incomplete ‘house training’ (i.e. urinates or defecates in the house regardless of the presence or absence of the owner)
6. At least one of the following signs exhibited during an eliciting context: Inappropriate urination, Inappropriate defecation, Destructive behaviour, Excessive vocalisation	6. Received treatment with any of the following psychoactive medications within 1 month before this visit: Tricyclic antidepressants (e.g. amitriptyline); Monoamine oxidase inhibitors (e.g. selegiline); Carbamazepine; Serotonin reuptake inhibitors (e.g. clomipramine, fluoxetine, fluvoxamine); Propanolol; or psychoactive herbal product e.g. St. John’s Wort (*Hypericum perforatum*)
7. At least one of the defined separation signs in at least half of the eliciting contexts	7. History of aggressive behaviour shown towards people, which puts humans at risk of physical injury
	8. Synthetic pheromone product (e.g. DAP) being used in the home for any reason.
	9. Other diagnosed behavioural problems or any other suspected behavioural problem.

Inclusion criteria 5, 6 and 7 were only applicable to the Clinical Group.

For the control group, pet dogs were recruited on the basis of an absence of any SRP-related incidents or any other anxiety problem. These dogs were recruited via local advertisement within the University of Lincoln to both university students and staff. The owners had to complete a questionnaire about their dogs’ behaviour. Only if the dogs fulfilled inclusion criteria 1–4 in Table 1, above and met none of the exclusion criteria, were they included in the study. Eight dogs of various breeds were recruited. One dog was subsequently excluded due to low food motivation. This left 3 males (all neutered) and 4 females (3 neutered and 1 intact). The average age of the dogs was 4.9 years old (range 7 months and 11.5 years old), and they had been owned for, on average, 4.6 years (range 7 months - 10 years). Their average body weight was 19.6 kg (range 10.5 - 27 kg).

### Cognitive Bias (CB) test

**Figure 1:**
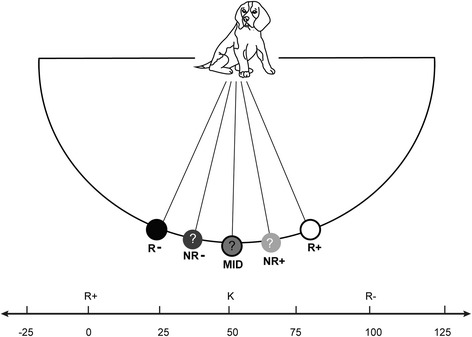
**Schematic representation of the Cognitive Bias test layout.** The dog, starting from the start position, moves to one of five locations on an arc 3–4 metres away, marked by the presence of a food bowl (only present at one location at any one trial). R-: Non Food position (negative location), NR- : Near Non Food ambiguous position, MID: Middle ambiguous position, NR+: Near Food ambiguous position and R+: Food position (positive location). Different distances were used on the basis of available space, but were consistent within subjects.

The dogs underwent an assessment of cognitive judgement bias before and during treatment. The CB test was based on Mendl et al. [18]. The dogs were trained to discriminate a food bowl placed in one of two different locations, one with food and one without. The locations were the same distance from the start position as illustrated in Figure 1. Dogs were trained such that when the bowl was placed to one side of the room, (the positive location, R+), it would contain food, and when it was placed on the other side, (the negative location, R-), the bowl would be empty (Figure 1). Following a random allocation procedure, the positive location was to the right for three dogs from the clinical group and for four dogs from the control group, whereas for the remaining two dogs from the clinical group and three dogs from the control group it was to their left. The learning phase was completed after a minimum 15 trials, and determined to be complete when, for three consecutive negative trials, their average absolute speed (m/s) towards the negative location was less than the average speed to the positive location. Dogs were then tested with three ambiguous probes positioned between the learned ‘reference’ locations: (1) midway (MID) between the positive and the negative locations, (2) midway between the positive and MID probe locations (NR+); (3) midway between the negative and MID probe locations (NR-) (Figure 1). All ambiguous locations were unrewarded (Additional file 1). The testing phase contained nine exposures to the three ambiguous probes in total, i.e. three for each probe, interspersed between exposures to the learned ‘reference’ locations. In total, the testing phase included 40 trials, as testing started with a probe trial.

### Study design

In accordance with other clinical trials for this behaviour problem, baseline measures were gathered over a two week period before the onset of 8 weeks of treatment [14-16]. For the control group, the study lasted only 6 weeks as the dogs did not require either the pre-enrolment visit which was used to instigate measurement of baseline severity of SRP nor the follow-up at the end of the treatment period (Table 2). Three CB tests were performed by both groups with the same interval between tests +/− 2 days (Table 2).

**Table 2:** **Study schedule of events for the clinical and control groups**

**Time and action**	**Pre-enrolment visit**	**Baseline**	**+2 weeks**	**+4 weeks**	**+6 weeks**	**+8 weeks**
**Clinical group**	Pre-screening for SRP	CB1	CB2	Telephone FoU-1	CB3	Telephone FoU-2
		3-part Question.	3-part Question.	3-part Question.	3-part Question.	3-part Question.
		Review of dog’s diary				Review of dog’s diary
**Control group**	Not required	CB1	CB2	Not required	CB3	Not required
		3-part Question.			3-part Question.	
		Pre-screening for SRP			Post-screening for SRP	
	**Enrolment period**		**Treatment Period**			

SRP: Separation related problems, CB: Cognitive Bias test, Question: Questionnaire, FoU: Follow-up.

### Clinical group

At the first visit (Pre-enrolment) the owners completed a pre-screening questionnaire about their dog and provided a behaviour history of their dog’s separation behaviour (approximately duration 1–1.5 hours) (Table 1). The inclusion and exclusion criteria were reviewed and a physical examination was performed on the dog by a veterinarian specialising in behavioural medicine (CK), with diagnoses confirmed by the supervising behaviour specialist (DM). Suitable dogs were then pre-enrolled for the study and the owner completed an informed consent form. Then, the eliciting contexts (EC) were identified, i.e. times when the owner left the dog on its own, e.g. for work or to go shopping, which might evoke a SRP-related response [13]. For the following two weeks, the owners recorded on individualised recording sheets, for each EC, the specific separation-related behaviours (SR-BEH) of the dog (dog’s diary). A similar record was kept for the last two weeks of the treatment period for comparative purposes. At the baseline visit, if the dogs had shown more than eight SR-BEH during the two-week pre-treatment period, they were enrolled as cases.

At the baseline visit, dogs were assessed for the first time with a cognitive bias test (CB1) and the owners completed a 3-part questionnaire about their dog’s tendency for attachment towards them, the SR-BEH it showed, and provided a “separation anxiety global score” for the SRP (SAGS) (Additional file 1). The same questionnaire was completed during the telephone follow-up sessions. All questionnaire measures related to the owner’s assessment of the frequency or intensity of behaviours over the previous two weeks rather than on any specific day. A standardised behaviour modification plan (Table 3) was explained to the owners and Fluoxetine (Reconcile™, Elanco, 1-2 mg/kg by mouth, once daily) was provided at this time. The next two visits (week +2 & week +6) were during the treatment period and were booked at this time for two and six weeks after baseline respectively, when the owners again completed the same 3-part questionnaire and the dog was again CB tested.

**Table 3:** **Summary of behaviour modification plan, used in treatment of cases**

**AT HOME**	**Interact with dog only at your initiative and when the dog is relaxed.**
	Praise the dog when it is relaxed.
	Gradually teach your dog to stay calm and to be alone i.e. have it sit, lie down, or stay in places as you move away while gradually increasing the distance and time from the dog.
	Give departure cues at times other than departure.
	Praise calm behaviour if appropriate.
**BEFORE LEAVING**	Show complete indifference to the dog for 20 to 30 minutes prior to going out.
	As you leave, you may give a special toy or a treat to distract the dog and remove the item on return - make this something special, like a food-filled treat, so that your leaving is associated with something positive.
	Do not physically or verbally interact with the dog just before leaving.
**WHEN RETURNING**	Ignore the dog’s excessive greeting until he is quiet and relaxed.
	Interact with your dog only on your initiative and only when he/she is quiet.
	Reward calm behaviour.
	Do not reprimand dog for destructive behaviour or for urinating or defecating in the house.

The same medication and dose (Reconcile™, Elanco, 1-2 mg/kg by mouth, once daily) as has been used previously in a placebo controlled clinical trial was used [19]. A placebo tablet treated group was scientifically unnecessary for the aims of the present study, which was to investigate whether clinical improvement of SRP-afflicted dogs during treatment resulted in measurable changes in CB tests and not to determine the effect of the different treatment components. For the same reason, owners were not blinded as there was no placebo group and also because the owners’ perception of their dog’s behaviour, recorded via questionnaire, could be compared with the more objective CB tests. A control ‘no treatment’ group tested at similar intervals was used to control for time related effects and the effects of repeat testing with the CB tests.

### Control group

Similar to the clinical group, at the first enrolment visit the owners completed a pre-screening questionnaire about their dog and provided a behaviour history about the dog’s separation behaviour. The inclusion and exclusion criteria were reviewed and a physical examination was performed on the dog by the same veterinarian as the clinical subjects. Then, potential eliciting contexts were identified, i.e. times when the owner left the dog on its own, e.g. during work or shopping, which might theoretically evoke a problem response. Retrospectively for the previous two weeks, the owners recorded for each EC the (SR-BEH). If the dogs had shown only one or no SR-BEH during the two-week pre-treatment period, they were enrolled onto the study and the owner completed an informed consent form.

Then the owners completed the same 3-part questionnaire about their dog’s tendency for attachment towards them and SR-BEH, and a global score for the SR-BEH (SAGS). Dogs that could complete the CB test procedure and fulfilled the EC’s frequency requirements (Table 1) were included in the study data set. Owners were also asked to report any behaviour changes in their dog during the assessment period, in order to avoid including dogs with SRP in the control group.

The dogs of the control group performed the CB test on baseline, and 2 and 6 weeks later, on weeks +2 and +6 respectively. They were tested in equivalent conditions to the dogs in the clinical Group.

### Statistical analysis

The age, the duration of their ownership and the body weight of subjects in the clinical and control groups were compared using a Mann–Whitney U test. Due to the different sizes and possible food motivations of the dogs, the running speeds of each dog was adjusted as recommended by Mendl et al. [18], to take into account the mean speed of subjects to get to the positive and negative locations at the end of training. Adjusted speed during testing was calculated as follows:

$$ \begin{array}{l}\mathbf{Adjusted}\ \mathbf{speed}\kern0.5em =\kern0.5em \Big[\left(\mathrm{mean}\ \mathrm{speed}\ \mathrm{t}\mathrm{o}\ \mathrm{positive}\ \mathrm{location}\ \hbox{--}\ \mathrm{speed}\ \mathrm{t}\mathrm{o}\ \mathrm{probe}\ \mathrm{location}\right)\\ {}\times \kern0.5em \mathrm{mean}\ \mathrm{speed}\ \mathrm{t}\mathrm{o}\ \mathrm{negative}\ \mathrm{location}\times 100\Big]\\ {}/\Big[\left(\mathrm{mean}\ \mathrm{speed}\ \mathrm{t}\mathrm{o}\ \mathrm{positive}\ \mathrm{location}\ \hbox{--}\ \mathrm{mean}\ \mathrm{speed}\ \mathrm{t}\mathrm{o}\ \mathrm{negative}\ \mathrm{location}\right)\\ {}\times \kern0.5em \mathrm{speed}\ \mathrm{t}\mathrm{o}\ \mathrm{probe}\ \mathrm{location}\Big].\end{array} $$

Consequently, if the speed to the probe location was equal to the mean speed to positive (food) location, the adjusted score was 0, and if the speed to probe location was equal to the mean speed to negative (non-food) location, the score was 100 (Additional file 1). Only adjusted speeds towards the ambiguous probes are subsequently assessed and reported.

The data were analysed using SPSS 17.0. Normality was assessed using Kolmogorov-Smirnov tests, and, as some factors were significantly different to normal (P < 0.05), all data were analysed using non-parametric statistics.

A statistical plan was formulated to accommodate the relatively small sample size, (we used 12 dogs in total, compared to 9 similar studies testing cognitive bias in dogs, rats, starlings and sheep that used from 6 to 32 animals (mean number of subjects: 19.9) [18,24-31], and consideration that a Bonferonni correction would be too conservative and likely to result in Type II errors [32]. As a precaution against the risk of Type I errors, the statistical plan prioritised testing of the primary hypotheses of interest (differences at baseline and time of maximum clinical effect i.e. week 6). The effect size (*r)* was calculated in each instance [33]. Subsequent data exploration was performed, conditional upon the outcome of these primary findings.

The first priority was to establish whether the clinical group differed significantly to the control group in their CB performance towards ambiguous probes at baseline, as would be expected given the results of Mendl et al. [18], (Mann–Whitney U-test). Where differences between groups were found, these were evaluated in line with the prediction that the clinical group should show a negative affective bias.

The next analysis compared the performance of subjects in CB tests between the clinical and control groups at week +6, using Mann–Whitney tests. In order to minimise the risk of Type II errors, this considered only the data relating to those probes which had been found to have been significantly different between groups at baseline. On the basis of these results, a similar assessment was made considering data from week +2 of treatment, in order to help identify the timescale of any change. After this, we compared within-groups over time, but for all ambiguous probes using Wilcoxon tests.

Only after these analyses were complete, was the consistency of the non-significant differences between groups at baseline assessed. In this way priority was given to the effect of treatment on those measures of most potential value first, reducing the risk of Type II errors from multiple testing.

The clinical assessment data were used to explore the convergent validity of possible inferences about affective state from the results of these CB tests. Specifically, changes in the relative frequency of SRP within eliciting contexts, separation anxiety global score, separation anxiety behaviour scores (the sum of individual problem behaviours shown: inappropriate urination, inappropriate defecation, destructive behaviour and excessive vocalisation) and attachment towards owner score at baseline versus weeks +2 and +6 were assessed for their significance (Wilcoxon signed rank test). Correlations between all measures were then explored using Kendall’s tau.

## Results

### Between-group analysis – clinical group and control group

Dogs in the clinical and control group did not show any statistical difference in their age, the duration of their ownership and their body weight (U = 16.5, Z = −0.163, p = 0.88; U = 11, Z = −1.061, p = 0.343, U = 13.5, Z = − 0.651, p = 0.53 respectively).

Initially, the clinical group’s baseline score to the NR- position was significantly slower than that of the control group U = 5, *z* = −2.03, *p* = 0.042, *r* = −0.59 (Table 4). Specifically, the clinical group’s score (Median = 68.9) tended to be relatively closer to the negative (R-) position (Median = 100), and the score of the control group (Median = 21.2) relatively closer to the positive (R+) position (Median = 0). There was no significant difference between the two groups in the baseline median speed towards the MID and NR+ probes (MID *U* = 11, *z* = −1.06, *p =* 0.291, *r = −*0.31, NR+ *U* = 9, *z* = −1.38, *p* = 0.167, *r* = −0.4 respectively (Table 4).

**Table 4:** **Median adjusted speeds with fastest and slowest speeds in parentheses toward ambiguous probes at baseline and during treatment**

**Probe**	**Group**	**Baseline**	**Week +2**	**Week +6**
**MID**	**CLINICAL GROUP**	34.0^b^ (2.1, 82.2)	4.6 (−11.3, 139.6)	2.5^b^ (−5.3, 7.0)
	**CONTROL GROUP**	13.3^d,e^ (6.1, 40.6)	53.1 ^d^ (22.7, 116.4)	81.9^e^ (24.5, 221.8)
**NR+**	**CLINICAL GROUP**	11.8^c^ (−0.1, 61.5)	0.4 (−21.2, 11.0)	0.8^c^ (−3.8, 1.0)
	**CONTROL GROUP**	3^f,g^ (0.1, 23.7)	12.7 ^f^ (4, 92.3)	21 ^g^ (3.9, 67.1)
**NR-**	**CLINICAL GROUP**	68.9^a^ (39.9, 141.9)	56.9 (6.88, 157.7)	52.3 (23.4, 100.0)
	**CONTROL GROUP**	21.2^a,h,i^ (7.5, 84.5)	59.6^h^ (22.1, 584.6)	72.1^i^ (62.9, 129)

0 = Adjusted Speed towards Food Position and 100 = Adjusted speed towards Non-food position.

Values with the same superscript letter were found to be significantly different.

**Figure 2:**
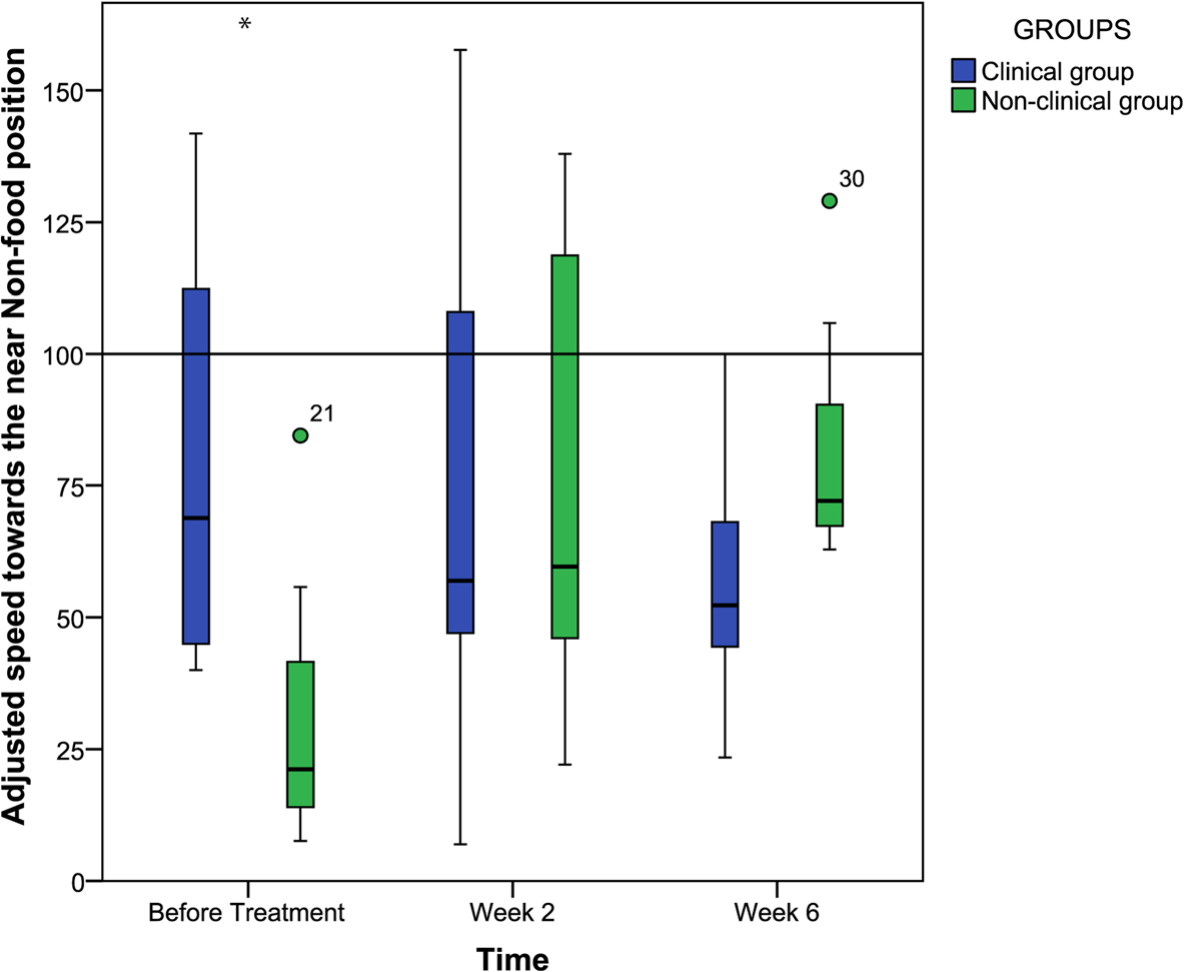
**Adjusted speeds of the two groups towards the NR- position.** (0 = Adjusted Speed towards Food Position and 100 = Adjusted speed towards Non-food position). * indicates a significant difference between the two groups at this time.

The effect of treatment on response to just the NR- probe was therefore next compared between the two groups in accordance with the statistical analysis plan outlined above. At week 6 there was no significant difference between the treatment and control groups in their response to the NR-probe (*U = 7, z = −1.71*, *p* = 0.11, *r* = −0.49), and there was also no significant difference between the two groups at week 2 (*U = 16*, *z* = −0.24, *p* = 0.88, *r* = −0.07). Thus, the two groups started the study differing in their response to the NR- probe stimulus at baseline but this difference had disappeared by week +2 and continued to be absent in week +6 of treatment (Figure 2).

### Within-group analysis - clinical group

The median score of the clinical group towards the MID position was significantly faster at week 6 than at baseline (Table 4, *z* = −2.023, *p* = 0.043, *r* = −0.9). The median response to the NR+ position was also significantly faster at week 6 compared to baseline (Table 4, *z* = = −2.023, *p* = 0.043, *r* = −0.9). However there was no significant change in response to the NR- position (*z* = −0.674, *p* = 0.5, *r* = −0.30) for the same comparison.

Comparisons between the baseline and week +2 did not show significant differences for any of the three ambiguous probes, although the response to the MID probe was borderline (NR+: *z* = −1.21, *p* = 0.23, *r* = −0.54; MID: *z* = −0.67, *p* = 0.05, *r* = −0.3; and NR-: *z* = −0,41 *p* = 0.69, *r* = −0.18). Thus, changes in response towards the MID and NR+ positions were only evident after six weeks of treatment, with no changes for the NR- probe.

### Within-group analysis – control group

There was a significant effect of repeated CB testing on the Control group, with dogs moving significantly more slowly to all three ambiguous probes at both week +2 (MID: z = −2.197, p = 0.028, r = −0.83; NR+: z = −2.366, p = 0.018, r = 0.9; NR-: z = −2.366, p = 0.018, r = 0.9) and week +6 (MID: z = −2.366, p = 0.018, r = 0.9; NR+: z = −2.366, p = 0.018, r = 0.9; NR-: z = −2.197, p = 0.028, r = −0.83) compared to baseline (week 0) performance (Table 4).

### Changes in clinical signs following treatment – clinical group

The four clinical measures of treatment efficacy showed a statistically significant improvement when comparing the two weeks preceding treatment (assessment at baseline) to the last two weeks of the treatment (assessment at week +8 of treatment). The proportion of eliciting contexts in which SRP were expressed decreased from a median of 0.9 to 0.14 (*z* = −2.032, *p* = 0.042, *r* = −0.9). The separation anxiety global score fell from a median of 3 (severe) to 0 (absent) (*z* = −2.041, p = 0.041, r = −0.91). The sum of individual behaviours (inappropriate urination, inappropriate defecation, destructive behaviour and excessive vocalisation) fell significantly from a median of 4 to 0 (*z* = −2.060, *p* = 0.039, *r* = − 0.921). Scores for attachment towards the owner fell from a median of 15 to 4 (z = −2.032, *p* = 0.042, *r* = −0.909).

### Correlation between clinical measures – clinical group

The clinical measures of separation anxiety severity: frequency of the eliciting contexts; separation anxiety-related behaviour scores; separation anxiety global score; and attachment behaviour scores were all significantly correlated (*p* < 0.01 in all instances, Table 5).

**Table 5:** **Correlations (Kendall’s tau) between clinical measures and associated statistical significance (2-tailed)**

		**EC**	**SABS**	**SAGS**
**SABS**	Correlation coefficient	0.880		
	Sig.	0.001		
**SAGS**	Correlation coefficient	0.845	0.716	
	Sig.	0.001	0.001	
**ATTACH**	Correlation coefficient	0.754	0.642	0.858
	Sig.	0.005	0.002	0.000

Eliciting Contexts Frequency (EC), Separation Anxiety Behaviour Scores (SABS), Separation Anxiety Global Score (SAGS) and Attachment score (ATTACH).

### Correlation between CB and clinical measures – clinical group

Within the group receiving treatment, all clinical measures of SRP correlated significantly with the adjusted speed towards MID (recorded at baseline, week +2 and week +6), but none with the adjusted speed towards the NR- probe. Adjusted speed towards the NR+ probe correlated significantly with separation anxiety global score and attachment score, but not with eliciting context frequency and separation anxiety behaviour scores (Table 6).

**Table 6:** **Correlations (Kendall’s tau test) across time between median speeds and clinical measures with associated significance (2-tailed)**

		**EC**	**SAGS**	**SABS**	**ATTACH**
**MID**	Correlation coefficient	0.584^*^	0.534^**^	0.403^*^	0.571^**^
	Sig.	0.020	0.009	0.047	0.004
**NR+**		0.449	0.408*	0.114	0.411*
	Sig.	0.072	0.046	0.576	0.039
**NR-**		0.180	0.345	0.217	0.270
	Sig.	0.472	0.091	0.285	0.174

* = P<0.05, ** = p<0.005.

Eliciting Contexts Frequency (EC), Separation Anxiety behaviour scores (SABS), Separation Anxiety Global Score (SAGS), Attachment score (ATTACH).

Correlations across time between the median speeds of clinical subjects (on baseline, week +2 and week +6) and the clinical measures during the analogous period of study (N = 15). Only for the EC, correlations between EC and median speeds were based on data from baseline and week +6 (N = 10).

## Discussion

These results show, for the first time, that clinical treatment of a naturally occurring negative affective state and associated behaviours in a non-human species can produce a significant shift in cognitive bias as well as a large improvement in clinical behaviour measures as determined by statistical effect size. The difference evident at baseline in CB testing between treatment and control groups (i.e. response to NR- probe) disappeared by week 2 of treatment and remained absent after 6 weeks. It seems that in normal (i.e. Control) dogs that the running speed towards this probe reduces with repeat testing, whereas in the Clinical group, such a reduction in speed over time is not seen. We interpret this as an indication that the intervention on the Clinical group reduced the negative bias that develops towards this probe over time. This change together with the increased speed of Clinical group dogs to the two other probe locations across the study period meant that, in effect, the clinical population normalized with regards to the CB test over the course of the study. The four clinical measures used to assess the severity of SRP in this study showed convergent validity between themselves, and also demonstrated a statistically significant improvement with treatment implementation, reinforcing the findings from the CB tests on these subjects. The clinical group, not only showed signs of clinical improvement but also increased running speed to the MID and NR+ probes, which is consistent with the “optimistic” affective bias predicted for an antidepressant. However, we acknowledge that at present, it is not conclusive which aspect of the treatment plan, fluoxetine, the behaviour modification plan or the combination of the two, evoked the observed shift in cognitive bias. This question could be addressed in the future with the use of a double blind placebo controlled study using a similar protocol. This does not detract from the importance of the current study whose aim was to examine, as a critical first step, the association between the dogs’ behavioural improvement during treatment and possible changes in their underlying affective state, rather than make assumptions about this on the basis of superficial behavioural changes which could have an alternative interpretation and markedly different welfare implications. Subsequent studies will specify the impact of each individual component of the treatment plan.

A previous study which tested dogs housed at a rescue shelter found that dogs in a predictive test of SRP appeared to show a more ‘pessimistic’ cognitive bias towards the MID probe, but not towards the NR+ and NR- probes [18]. By contrast, in our study, before treatment implementation, dogs with clinically diagnosed SRP showed a ‘pessimistic’ bias to only the NR- probe compared to controls. Both studies agree that dogs with SRP appear to have a pessimistic cognitive bias, suggestive of a background negative affective state, and this adds to the evidence that SRP is a serious welfare concern that needs addressing. However, the difference between the studies in the particular probes which appear to reveal this effect (MID vs NR-) may reflect differences in either the population tested (clinical cases in the current study, versus shelter dogs undergoing a predictive test in the case of Mendl et al. [18]) or test environment (home versus shelter). It has been proposed that different types of affective state might result in differentiable performances towards specific probes, i.e. anxiety may result in changes largely towards the NR- probe while depression-like states would largely induce changes towards the NR+ probe [16,17]. In which case, our observed differences at the probe located closest to the unrewarded/negative reference location (NR-) at baseline suggest that the dogs in our clinical group experienced an increased sensitivity to aversion, a feature of many anxiety disorders. However, the response to treatment within the group was most marked towards the NR+ location and this is consistent with fluoxetine primarily being an antidepressant rather than tranquilizer.

The control dogs showed evidence of learning that the probes were unrewarded during repeat testing, as has been previously found in sheep [24]. As our control dogs did not have any obvious reason to change affective state during the study, and there were no notable changes in their environment according to their owner, it seems reasonable to suppose that the reduction in speed to the ambiguous probes reflects learning that these probes were unrewarded. In contrast, we observed that our clinical dogs increased their speed to the probes over time. An affective explanation for this result would be that the dogs become increasingly ‘optimistic’ during treatment. Nonetheless, it is important to consider alternative, non-affective explanations, and one obvious consideration relates to increased food motivation as a result of the medication. However, a side-effect of fluoxetine is to suppress appetite [20], and so the side effects of fluoxetine would, if at all, be expected to reduce running speed towards the probes (i.e. the opposite effect to what was found in this study), therefore the observed increase in running speed over time cannot obviously be accounted for by an increased motivation for food as a result of the medication.

In the clinical group, daily administration of fluoxetine in combination with a behaviour modification plan, significantly improved all clinical measures used for monitoring the dog’s behaviour, confirming other studies [19]. This improvement correlated (Table 6) with an increase in the speed with which clinical dogs ran towards the MID probe, suggesting that the behavioural improvement in the SRP was also associated with an improvement in the dog’s underlying affective state. Again, this finding is contrary to a potential alternative non-affective explanation, that the beneficial behavioural effects of serotonergic agents on problem behaviour in companion animals may be due simply to their broad inhibitory effects on behaviour [34]. If this were the case, then although clinical signs may improve (e.g. less separation related behaviours), response speed in the CB test would be expected to decrease rather than increase following administration of fluoxetine. It is worth noting that citalopram, another selective serotonin reuptake inhibitor (SSRI) agent has been shown to increase memory of positive materials (e.g. positive personality traits) in healthy human volunteers [35], and this positive effect on affective state seems the most parsimonious potential explanation for the effect seen. Serotonin is an important neurotransmitter that innervates the amygdala directly [36] and indirectly affects reward seeking behaviour [37]. Furthermore, fluoxetine, as a 5HT2C antagonist, also has disinhibitory effects on the release of norepinephrine and dopamine within the prefrontal cortex [36]. Thus, fluoxetine may functionally reorganize the mesolimbic dopamine system, to increase reward seeking and sensitivity [37].

## Conclusion

Following treatment with a behaviour modification plan and fluoxetine, dogs with SRP not only improved their behaviour when alone but also showed an apparent improvement in the initial pessimistic affective state associated with the potential availability of rewards, at other times. Importantly, this is the first study to suggest that the clinical improvement of a spontaneously occurring behavioural problem is also associated with changes in affective state as determined by a CB test, and that SSRIs do indeed have an antidepressant effect on dogs.

## Additional file

Additional file 1:
**Additional methodological information.**
