# Supplementary material for: Dogs with separation-related problems show a “less pessimistic” cognitive bias during treatment with fluoxetine (Reconcile™) and a behaviour modification plan
Source: BMC Vet Res. 2015 Mar 28;11:80. doi: 10.1186/s12917-015-0373-1 (PMC4393593; doi:10.1186/s12917-015-0373-1)
Supplement: Additional file 1: — Additional methodological information. [file 12917_2015_373_MOESM1_ESM.doc]

**Additional File 1**

Additional File Table 1. Details of the dogs taking part in the study

|  | **Breed** | **Sex** | **Age (Years)** | **Weight (Kg.)** | **Ownership (Years)** |
| --- | --- | --- | --- | --- | --- |
| **Clinical group** | Cross Breed | Female (Neutered) | 6.5 | 11.0 | 6.0 |
|  | German Shepherd | Female (Neutered) | 4.6 | 30.7 | 0.2 |
|  | Labrador Retriever | Male (Neutered) | 2.0 | 28.0 | 0.6 |
|  | Cross Breed | Male (Neutered) | 5.2 | 22.0 | 4.3 |
|  | Cross Breed | Male (Intact) | 2.8 | 21.5 | 1.0 |
| **Average:** |  |  | 4.2 | 22.6 | 2.4 |
| **Control group** | Cross Breed | Female (Neutered) | 0.6 | 27.0 | 0.6 |
|  | Basset Hound | Female (Neutered) | 10.0 | 23.0 | 10.0 |
|  | Labrador Retriever | Female (Neutered) | 2.0 | 24.0 | 2.0 |
|  | Cross Breed | Female (Intact) | 1.0 | 10.5 | 1.0 |
|  | Cross Breed | Male (Neutered) | 5.5 | 22.0 | 5.0 |
|  | Cross Breed | Male (Neutered) | 11.5 | 18.0 | 10.0 |
|  | Cross Breed | Male (Neutered) | 4.0 | 12.5 | 3.5 |
| **Average:** |  |  | 4.9 | 19.6 | 4.6 |

**Cognitive Bias**

1. **Introduction on Cognitive Bias**

At the beginning of each trial, the dog was left off lead to explore the area. Then the owner with the dog, or the researcher with the dog, in case of the five dogs in control groups, sat in a chair to one side of the room and held the dog gently by its collar, to stop the dog from moving away. The position of the dog was marked by a spot on the floor. On the other side of the room, 3m or 4m away from the dog was the researcher who placed the food bowl in the five predetermined positions, 2 training positions and 3 probes. The distances between the dog and the training/probe locations were the same. The same routine was followed in all the positions, food and non-food positions, so as not to allow the dog to learn the task from the researcher’s behaviour.

For all dogs with separation related problems and for two of the control dogs, the cognitive bias (CB) training/testing was carried out with the dog’s owner as the handler and one researcher for placing the food bowl. Five dogs in the control group were tested by two researchers. It has previously been reported that the presence or absence of their owner did not have any effect on dogs’ performance in a CB test [1]. One dog was tested outdoors, in a house environment, and the others indoors, in the demonstration room of Lincoln University. The testing room had dimensions approximately 12m x 13m and the outdoor space 4m x 4m. Each dog was tested in the same environment during all CB tests. As dogs’ attitude towards the ambiguous locations was evaluated in comparison with their baseline attitude, the most important factor was to keep the parameters for each dog consistent throughout the study.

1. **Recorded Measures**

Measures recorded in the CB tests included latency to approach each bowl, defined as the period of time when the dog was released until it put its nose in the bowl. The maximum period of time that the dog had to approach the food bowl was 30s. After that period the food bowl was removed and the dog was recalled back to its owner for the next repetition. The handler, owner or researcher, was the same for each dog during the whole study.

**Training and testing procedures**

In the beginning of the test, the dogs were taught to discriminate between the unrewarded ‘negative’ (R-) and the rewarded ‘positive’ (R+) probes, through repeated training trials. At the start of training, each dog received two consecutive positive trials followed by two negative trials. After that, positive and negative trials were presented in a pseudorandom order, with no more than two trials of the same type being presented consecutively. The training trials continued until the dogs’ mean speed towards the negative position was less than its mean speed to positive position for three consecutive trials. Once criterion was achieved, training continued until the dogs had performed two consecutive positive (R+) repetitions, so that the trials experienced immediately prior to the test trials where the same for all dogs. During testing, dogs were exposed to three ambiguous probes locations, positioned between R- and R+, with one in the middle position (MID), one (NR-) between R- and MID and one (NR+) between R+ and MID. All the tested locations were unrewarded.

Each dog experienced each of the three ambiguous probes three times during the testing (nine probe exposures in total). Probe exposures were separated by four presentations of the trained locations (R+ and R-), with each test sequence starting and ending with a probe presentation. The probe trials were presented in the following order: MID, NR+, NR-, NR+, NR-, MID, NR-, MID, NR+ [2]. After all nine test trials had been presented a final trial was performed placing a bowl with food in the non-food location. The purpose of this was to check that dogs run slower to the non-food position because they learned the task and they were not relying on odour, visual or other cues.

**Attachment and Separation Anxiety Global Score**

The Attachment Score was composed of five questions about, how often the dog:

1. Follows the owners around the house?
2. Wants to sit in contact with the owner?
3. Becomes distressed when he/she cannot see the owner?
4. Becomes distressed when the owner prepares to leave?
5. Greets the owner excessively when owner returns?

The frequency of the behaviours was scored using a five-point scale:

0 = Never

1 = Rarely

2 = Sometimes

3 = Usually

4= Always

The separation anxiety global scores (SAGS) related to four behaviours:

1. Inappropriate urination
2. Inappropriate defecation
3. Destructive behaviour
4. Excessive vocalisation

The severity of the behaviours was completed using a five-point scale:

0 = Absent

1 = Mild

2 = Moderate

3 = Severe

4 = Very severe

The sum of the separation anxiety behaviour scores for a period of two weeks is equally weighted e.g. i) Inappropriate urination = Sometimes ( 2 ), ii) Inappropriate defecation = Never ( 0 ), Destructive behaviour = Rarely ( 1 ), Excessive vocalisation = Usually ( 3 ). Hence, the Separation anxiety global score for this period would be: 2 + 0 + 1 + 3 = 6.

The individual scores for were summed and one score for each two week period was obtained for the attachment and one for the separation anxiety behaviours

**Adjusted Speed**

We modified the formula used by Mendl et al. [2] so that it was based on speed rather than latency values, so that differences in distance between subjects could be more fully acknowledged.

The adjusted score, based on the original formula [2] (Formula 1) = [(mean latency to ambiguous probe – mean latency to positive location) x 100] / [(mean latency to negative location – mean latency to positive location)].

We replaced latency with speed, using the calculation: distance / latency, such that the adjusted score became based on speed (Formula 2) = [(mean speed to positive location – mean speed to ambiguous probe) x mean speed to negative location x 100] / [(mean speed to positive location – mean speed to negative location) x mean speed to ambiguous probe].

As such, if the mean speed to ambiguous probe (Formula 2) is equal to the speed to positive (food) location, the adjusted score is 0, and, on the other hand, if the mean speed to ambiguous probe is equal to the speed to negative (non-food) location, the score is 100. In a scale from 0 to 100, the zero point is the score to food location (F) and the 100 point is the score to non-food location (NF). The same adjusted score results are achieved using Formula 1 and latencies.

**Example:** If a dog takes an average of 2 seconds to run to the positive location which is at a distance of 4 metres, then the mean speed to the positive location is 4 metres / 2 seconds = 2 m/s. If the same dog takes an average of 8 seconds to reach the negative location, the mean speed to the negative location is 4 metres / 8 seconds = 0.5m/s. For the ambiguous probe K, if the dog takes an average 5 seconds to reach it, then the mean speed to the ambiguous probe is 4 metres / 5 seconds = 0.8 m/s.

According to Formula 1(latency): the adjusted score for the ambiguous probe K is = [(5 seconds – 2 seconds) x 100] / [(8 seconds – 2 seconds)] = 50, which means half way between the food and the non-food latency.

According to Formula 2 (speed): the adjusted score for the ambiguous probe K is = [(2m/s – 0.8m/s) x 0.5m/s x 100] / [(2m/s – 0.5m/s) x 0.8] = 50, which means half way between the food and the non-food speed (Additional File Figure 1).

**Additional File Figure 1. Scale of Adjusted Speed**

**
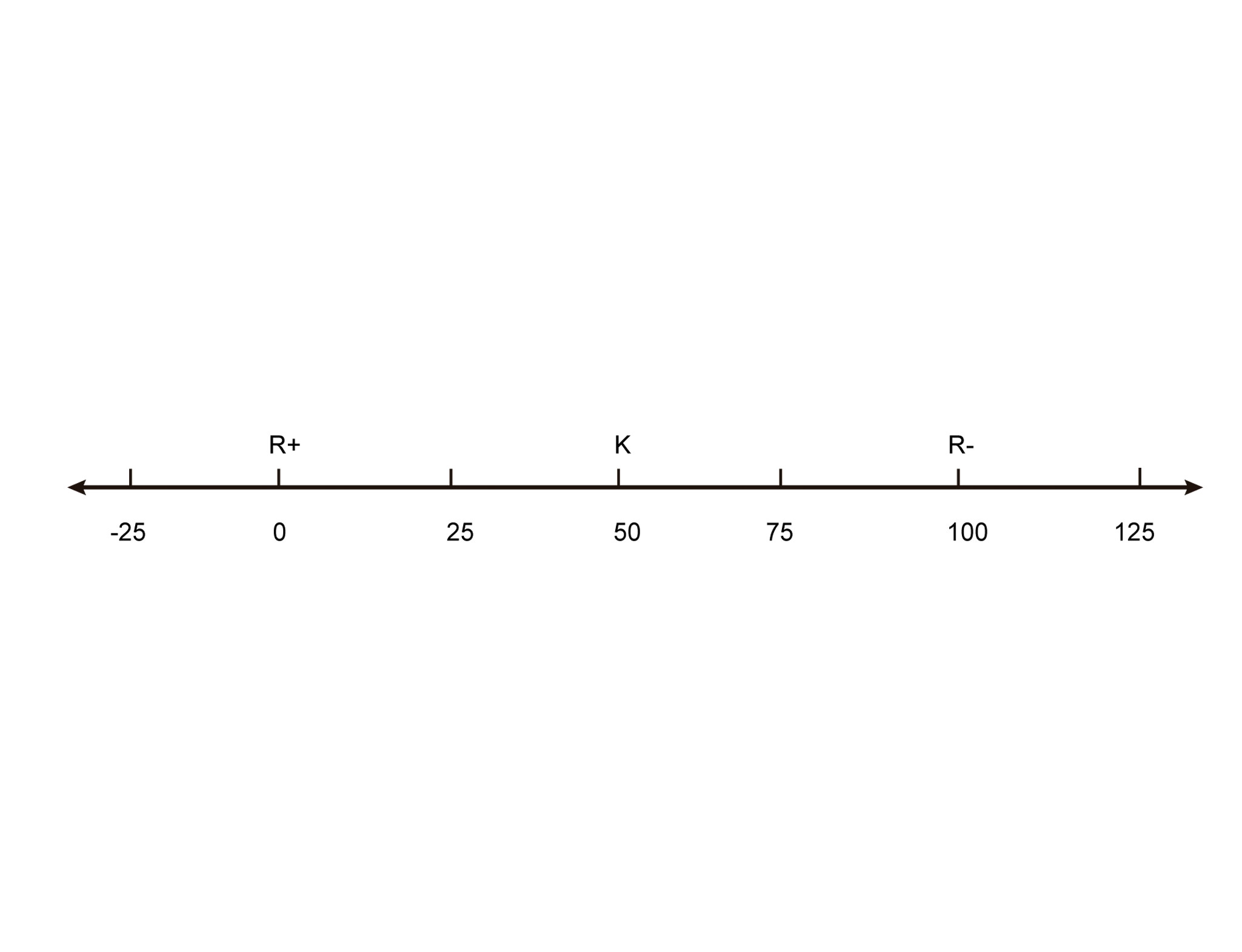
**

On a scale from 0 to 100, the zero point is the score to food probe (R+) and the 100 point is the score to the non-food probe (R-). Thus, if a dog runs with an average speed of 2 m/s to the food location and average speed of 0.5 m/s to non-food location, then it has a mean speed to reach the K probe location of 0.8 m/s, this would give an adjusted score = {(2-0.8) x 0.5 x 100} / (2-0.5) x 0.8= 50. Dogs that run on average faster to a probe location than to the positive probe location could have negative adjusted scores and dogs that run slower on average to the negative location could have scores greater than 100.

**References**

1. Müller C. A., Riemer S., Rosam C. M., Schößwender J., Range F. & Huber L: **Brief owner absence does not induce negative judgement bias in pet dogs.** *Anim Cogn 2012,* **15(5):** 1031-1035.
2. Mendl M, Brooks J, Basse C, Burman O, Paul E, Blackwell E, et al. **Dogs showing separation-related behaviour exhibit a ‘pessimistic’ cognitive bias.** Curr Biol. 2010;20:R839–40.
